# Fibres from flax overproducing β-1,3-glucanase show increased accumulation of pectin and phenolics and thus higher antioxidant capacity

**DOI:** 10.1186/1472-6750-13-10

**Published:** 2013-02-09

**Authors:** Wioleta Wojtasik, Anna Kulma, Lucyna Dymińska, Jerzy Hanuza, Jacek Żebrowski, Jan Szopa

**Affiliations:** 1 Faculty of Biotechnology, University of Wrocław, Przybyszewskiego 63/77, 51-148, Wrocław, Poland; 2 Department of Bioorganic Chemistry, Institute of Chemistry and Food Technology, Faculty of Economics and Engineering, University of Economics, Komandorska 118/120, 50-345, Wrocław, Poland; 3 Institute of Low Temperatures and Structure Research, Polish Academy of Sciences, Okolna 2, 50-422, Wrocław, Poland; 4 Faculty of Biotechnology, Centre of Applied Biotechnology and Basic Sciences, Rzeszów University, Rzeszów, Poland; 5 Linum Fundation, Stabłowicka 149-147, 54-066 Wroclaw, Poland

**Keywords:** Biopolymers, Fibres, Flax, *Linum usitatissimum*

## Abstract

**Background:**

Recently, in order to improve the resistance of flax plants to pathogen infection, transgenic flax that overproduces β-1,3-glucanase was created. β-1,3-glucanase is a PR protein that hydrolyses the β-glucans, which are a major component of the cell wall in many groups of fungi. For this study, we used fourth-generation field-cultivated plants of the *Fusarium* -resistant transgenic line B14 to evaluate how overexpression of the β-1,3-glucanase gene influences the quantity, quality and composition of flax fibres, which are the main product obtained from flax straw.

**Results:**

Overproduction of β-1,3-glucanase did not affect the quantity of the fibre obtained from the flax straw and did not significantly alter the essential mechanical characteristics of the retted fibres. However, changes in the contents of the major components of the cell wall (cellulose, hemicellulose, pectin and lignin) were revealed. Overexpression of the β-1,3-glucanase gene resulted in higher cellulose, hemicellulose and pectin contents and a lower lignin content in the fibres. Increases in the uronic acid content in particular fractions (with the exception of the 1 M KOH-soluble fraction of hemicelluloses) and changes in the sugar composition of the cell wall were detected in the fibres of the transgenic flax when compared to the contents for the control plants. The callose content was lower in the fibres of the transgenic flax. Additionally, the analysis of phenolic compound contents in five fractions of the cell wall revealed important changes, which were reflected in the antioxidant potential of these fractions.

**Conclusion:**

Overexpression of the β-1,3-glucanase gene has a significant influence on the biochemical composition of flax fibres. The constitutive overproduction of β-1,3-glucanase causes a decrease in the callose content, and the resulting excess glucose serves as a substrate for the production of other polysaccharides. The monosaccharide excess redirects the phenolic compounds to bind with polysaccharides instead of to partake in lignin synthesis. The mechanical properties of the transgenic fibres are strengthened by their improved biochemical composition, and the increased antioxidant potential of the fibres supports the potential use of transgenic flax fibres for biomedical applications.

## Background

Flax (*Linum usitatissimum*) has been a valued crop plant throughout human history. Its main products are its oil and fibres, which are used in the pharmaceutical, cosmetic, food, paper and textile industries. In Poland, where there is a good climate for the growth of flax, cultivation has been limited in recent decades due to the commercial competitiveness of cotton fibre and rapeseed oil. The renewal of its cultivation is significant because of the possibilities for its waste-free applications and its possible biomedical uses [1]. In parallel with this renewal, there has been a development of research into its genetic modification in order to improve the fibre and oil quality and increase the plants’ resistance to pathogen infections.

Unlike the fibres of cotton, which are composed exclusively of cellulose, flax fibre is characterised by the presence of antioxidants. This makes it more valuable, giving it the potential to be used not only for textile manufacture, but also for the production of flax dressings and sutures [1]. Two major factors limit the yield of good quality flax fibres: any decline in plant productivity caused by infections; and the dependence of the retting process on external and internal conditions, such as the weather, the lignification of the plant cell wall, and the pectin and hemicellulose contents of the fibre.

The flax retting process involves the separation of flax fibres from the epidermis and the cortical part of the stalk through pectin and hemicellulose degradation by microorganisms. The most common method of retting is the dew method, which involves flax decomposition in the field, with fungi and bacteria degrading the polysaccharides in the cell walls of the stalks [2].

Pectin is a polysaccharide with galacturonic acid residues constituting up to 70% of its overall structure. It consists of four pectin domains: homogalacturonan (HGA), rhamnogalacturonan I (RGI), rhamnogalacturonan II (RGII) and xylogalacturonan (XGA). Pectin plays a significant role in both plant physiology and plant defence against pathogen infection, as it constitutes a structural barrier to attack by fungi [3,4]. The quantitative and qualitative contribution of pectin to the construction of the plant cell wall depends on the plant species, so there are various possible consequences of modifications of pectin metabolism in various species [5].

Lignification of the cell walls in stalks is also a relevant issue for retting and thus for fibre quality. Flax intended for fibre production is harvested on day 107 of plant growth to prevent too great a degree of cell wall lignification. In order to improve the yield of flax, research is focused on generating transgenic flax plants that are more resistant to specific pathogens but characterised by unchanged or improved productivity relative to non-modified plants. Studies have shown that genetically modified flax with higher contents of certain constituents also has the potential to become a source of innovative products with biomedical properties.

Pathogen infections cause large loses in the yield from flax cultivation. Varieties of plants that are more resistant to infection have been generated through the introduction of genes for secondary metabolite synthesis or PR genes [6-11].

Plant β-1,3-glucanases are pathogenesis-related proteins classified as members of the PR-2 family. They are constitutively expressed at low levels, but their expression dramatically increases during infection. β-1,3-glucanase hydrolyses β-1,3-glucans, which are the main components of the cell wall of fungi. It acts in at least two different ways: directly, by degrading the cell walls of the pathogen; and indirectly, by promoting the release of cell wall-derived materials that can act as elicitors of defence reactions, stimulating the production of other PR proteins or low molecular weight antifungal compounds, such as phytoalexins [12,13]. By degrading callose, β-1,3-glucanases are also involved in various physiological and developmental processes, namely cell elongation [14], cell division [15], fruit ripening [16], fertilization [17], pollen germination and tube growth [18], bud dormancy release [19], microsporogenesis [20], somatic embryogenesis [21], seed germination [22,23] and flower formation [24,25]. Callose is a linear homopolymer that consists of glucose residues linked by β-1,3 binding with some β-1,6 branches, and it is widespread in higher plants [26].

Recently, in order to improve the resistance of flax to pathogen infection, we generated a transgenic plant that overexpresses β-1,3-glucanase. The resulting transgenic flax (named type B) was characterised by a threefold increase in resistance to *Fusarium oxysporum* and *F. culmorum* infection. *In vitro*-cultivated type B plants also displayed a significant decrease in the contents of carbohydrates, fatty acids and organic acids, and an increase in the levels of selected amino acids, polyamines and antioxidants [27].

The aim of this study was to investigate the influence of the constitutively expressed β-1,3-glucanase in flax type B on the biochemical composition of the cell wall and the mechanical properties of flax fibres in order to establish the usefulness of this transgenic plant for textile and pharmaceutical production. Of particular interest to us were the polysaccharide composition (pectin and hemicellulose) and the phenolic compound contents of the flax fibres. It was also important to analyse whether the overproduction β-1,3-glucanase could affect the quantity and the composition of the fibres obtained from the straw.

## Results

### Fibre quantity

**Figure 1:**
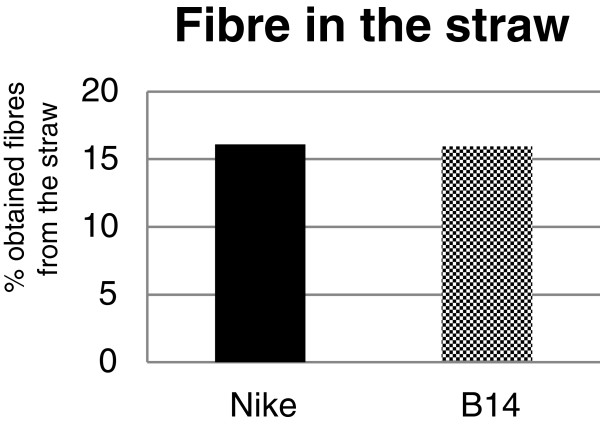
**Fibre content in the straw.** Plants of the transgenic flax line B14 and the control flax Nike were grown in a field and harvested on the 107^th^ day of their growth. The plants were retted for 40 days using the dew method. The percentage of fibre in the straw was determined. Black bar – control flax Nike. Checked black bar – transgenic flax B14. The precise method of retting is described in the Methods section.

The usability of flax fibre depends on the efficiency of the retting process. The transgenic flax line B14 and the non-transgenic flax line Nike gave similar yields of fibre from their straw (Figure 1). This indicates that the overexpression of the β-1,3-glucanase gene in flax had no influence on the quantity of the isolated fibre.

### Major cell wall structural components

Cellulose, hemicellulose, pectin and lignin are the major structural components of the plant cell wall. Cellulose, a linear polysaccharide consisting of β-1,4-linked D-glucose units, is the core of the plant cell wall, serving as a scaffold for the other cell wall components. Hemicellulose, a heteropolymer of polysaccharides, is divided into several classes: xyloglucans, glucomannans, glucuronoarabinoxylans and mixed linkage glucans [28]. Lignins are large, complex polymers of three principal alcohols: coniferyl, sinapyl and p-coumaryl [29].

**Figure 2:**
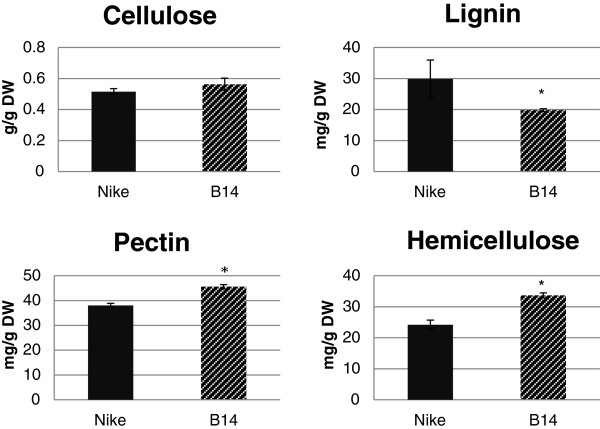
**Contents of major cell wall components.** The cellulose, lignin, pectin and hemicellulose contents were measured in the fibres of the transgenic flax line B14 (striped black bar) and the non-transgenic control flax Nike (black bar). Analyses were performed as described in the Methods section. Data represent the mean values ± SD from four independent experiments. The significance of the differences between the means was determined using Student’s t test (* - P < 0.05, ** - P < 0.01).

The amounts of those components were determined in fibres from transgenic flax line B14 and a control, non-transgenic flax line (Nike). The results are presented in Figure 2. The cellulose content was only slightly higher in fibres from B14 flax. However, the lignin level was significantly lower in fibres from B14 flax. The pectin and hemicellulose contents were higher in the fibres of the transgenic flax than in those of the control and these differences were statistically significant.

The transgenic flax plants overexpressing the β-1,3-glucanase gene were characterised by alterations in the composition of the cell wall. The higher cellulose content could indicate improved mechanical properties. The reduced lignin content could give better elastic properties and higher fibre flexibility. Additionally, the lower degree of lignification has a positive effect on the retting process, increasing its efficiency. However, the higher pectin content may have compensated for the lower lignin content and may be the reason that the efficiency of retting process was unchanged. No *in vitro* experiment on straw retting was performed, but the observed field retting time for the transgenic flax did not differ from that for the control.

### Infra-red spectrophotometry analysis

**Figure 3:**
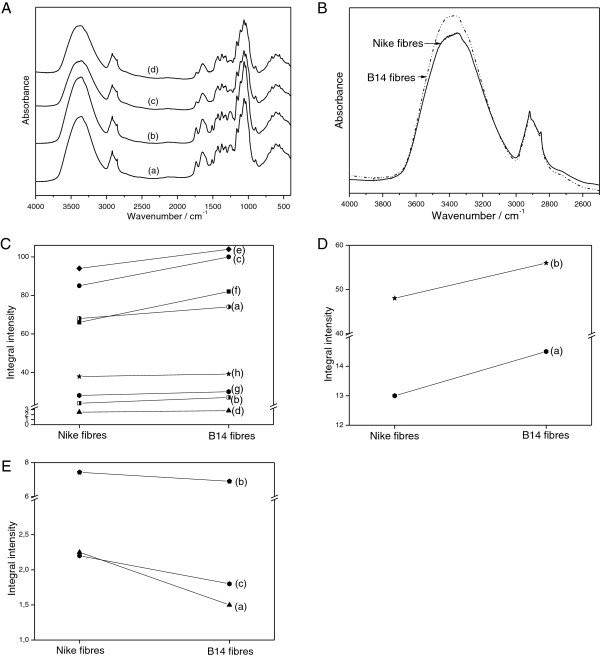
**IR spectrophotometry analysis of fibres from transgenic flax line B14 and non-transgenic flax Nike. A**. The IR spectra of Nike fibres (c) and B14 fibres (d). **B.** The IR spectra of the studied samples in the 4000–2500 cm^-1^ range. **C.** Differences in the integral intensities of the bands at 1429 cm^-1^ (a), 1367 cm^-1^ (b), 1318 cm^-1^ (c), 1236 cm^-1^ (d), 1025 cm^-1^ (e), 991 cm^-1^ (f), 898 cm^-1^ (g) and 665 cm^-1^ (h). **D.** Differences in the integral intensities of the bands at 1733 cm^-1^ (a) and 1609 cm^-1^ (b). **E.** Differences in the integral intensities of the band at 1335 cm^-1^ (a), 1263 cm^-1^ (b) and 1246 cm^-1^ (c). The mean square error for the values of the integral intensities of the bands after the deconvolution into Lorentzian components, obtained using the Origin software (OriginLab Corporation, USA), ranges from 0.00007 to 0.0007 for cellulose (Figure 3C), from 0.0001 to 0.0008 for pectin (Figure 3B) and from 0.000002 to 0.00009 for lignin (Figure 3E).

The IR spectra of the fibres from the Nike and B14 plants are presented in Figure 3A. Four characteristic ranges can be distinguished: 2500–4000, 1400–1800, 900–1400 and 400–900 cm^-1^. The main contours are similar to those reported for other flax fibres [30-33]. However, the relative intensities of several narrow lines that appear on the slope of these broad bands lead to an important conclusion on the chemical content of the transgenic flax. The IR spectra of the fibres from the control and genetically modified flax mainly consist of the bands that are characteristic for cellulose [30-36].

The broad absorption band at 3400 cm^-1^ corresponds to the stretching *v*(OH) mode of the free hydroxyl groups and those involved in intra- and inter-molecular hydrogen bonds [37]. The shape of this band is nearly the same for all of the studied samples, but the bands differ in terms of their absorption intensity (Figure 3B). The intensity of the contour in the range from 3000 to 3600 cm^-1^ clearly increases for fibres from B14 flax. This is probably caused by different conformations of the intra- and inter-molecular O-H···O hydrogen bonds in the B14 samples. The contours observed in the IR spectra of the flax fibres in the ranges 1500–1200 cm^-1^, 1200–950 cm^-1^ and 950–500 cm^-1^ are typical for flax cellulose with some amounts of lignin and pectin. The bands in these multiplets can be assigned to the vibration of δ_as_(CH_3_,CH_2_) at 1429 cm^-1^, δ_s_(CH_3_,CH_2_) at about 1372 cm^-1^, δ(CH) at 1319 and 1336 cm^-1^, *v*(C-C) and *v*(C-O) in the range 1200–1300 cm^-1^, δ(ϕ-OH) at 1163 cm^-1^, *v*_as_(C-O-C) in the range 1000–1110 cm^-1^, γ(CH) in the range 850–1000 cm^-1^, and δ(θ) in the range 500–720 cm^-1^. The spectrum in the 1200–1300 cm^-1^ range consists of a strong multiplet that is characteristic of in-plane bending vibrations of the hydrogen bond, δ(OH···O). Integral intensities of the component at 1236 cm^-1^ increase for fibres from B14 (8%) relative to fibres from Nike, suggesting an increase in the hydrogen bond number in the transgenic fibres. Other bands at 1318 and 1429 cm^-1^ respectively correspond to the δ(CH) + δ(OH) and δ_as_(CH_2_,CH_3_) vibrations. The comparison of the integral intensities of these bands gives the following relationships: for the 1429 cm^-1^ band I_B14 fibres_ > I_Nike fibres_ (24.2% difference) and for the 1318 cm^-1^ band I_B14 fibres_ > I_Nike fibres_ (17.6% difference; Figure 3C). The band at about 1367 cm^-1^ was described as cellulose and hemicellulose absorbance. Its integral intensities are higher for fibres from B14 (12.5% increase) than for those from Nike. The bands at 1025 and 991 cm^-1^ originate from the *v*(COC) vibrations of the β-1,4-glycosidic bond of the cellulose chains. The integral intensities analysed for these bands show the trend I_B14_ > I_Nike_. This difference is 10.6% (1025 cm^-1^) and 8.8% (991 cm^-1^; Figure 3C). The weak band at 898 cm^-1^ corresponds to vibrations of cellulose. The integral intensity for this band is slightly higher for fibres from B14 than for those from Nike (7.4%). The integral intensities of the band at 665 cm^-1^ corresponding to the γ(OH···O) vibration also change [37]. They are higher for fibres from B14 (5.3%). All these data suggest a higher amount of cellulose and hemicellulose in fibres from B14 than in those from Nike, which agrees with a chemical analysis data.

The IR bands in the 1800–1500 cm^-1^ range may be used to identify the changes in the pectin content in the fibres from the control and transgenic flax. IR spectra in this range can be deconvoluted into three Lorentzian components. The component at about 1737 cm^-1^ corresponds to the *v*_as_(COO) vibrations of the unconjugated carboxyl group of pectin. The integral intensity of this band fulfils the relationship I_Nike fibres_ < I_B14 fibres_, showing that transgenic flax B14 exhibits higher contents of pectin. This difference comes to 11.5% (Figure 3D). The position of the band at 1655 cm^-1^ corresponds to the *v*_as_(COO) vibration of the conjugated carboxyl group [38]. Its intensity slightly increases for fibres from transgenic flax. The third band, which appears at about 1605 cm^-1^, corresponds to the *v*_s_(COO) vibrations of the carboxyl group present in pectin. The strongest integral intensity is observed for fibres from B14 flax. The difference comes to 16.6% (Figure 3D). These data show that the content of pectin increases in fibres of transgenic flax line B14.

The IR bands in the 1300–1200 cm^-1^ range may be used to identify the changes in lignin content in the fibres from the control and transgenic flax. It can be deconvoluted into four Lorentzian components. The components at about 1263 and 1246 cm^-1^ correspond to lignin vibrations [37]. The integral intensities of these bands decrease for B14 fibres (7.13% and 14% for the bands at 1263 cm^-1^ and 1246 cm^-1^, respectively) suggesting a decrease in the lignin content in the B14 fibres (Figure 3E). Useful information can be obtained by comparing the integral intensities of the band observed at about 1325 cm^-1^, which corresponds to *v*(ϕ) (ϕ-ring) vibration. The intensities of this band fulfil the relationship I_B14 fibres_ < I_Nike fibres_. These data show that the lignin content is lower in the flax fibres of transgenic line B14 than in those from the control flax confirming the results obtained by chemical analysis.

### Mechanical properties

**Figure 4:**
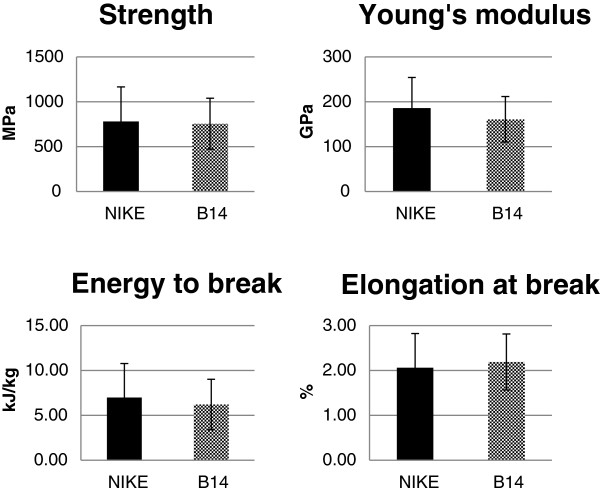
**Mechanical property analysis of flax fibres.** Four major mechanical parameters were measured for the retted fibres of transgenic line B14 (checked bar) and control flax Nike (black bar): tensile strength, tensile stiffness (Young’s modulus), energy to break, and relative extension at break. Analyses were performed as described in the Methods section. The mean values ± SD are presented (n = 30).

Overexpression of β-1,3-glucanase did not significantly alter the essential mechanical characteristics of the retted fibres, including their tensile strength, tensile stiffness, energy to break and relative extension at break (Figure 4). However, a statistically non-significant reduction was observed in Young’s modulus and the energy to break for fibres from transgenic flax line B14 relative to the values for the control, non-transgenic flax.

Despite some changes in the carbohydrate and lignin contents, the effect of introducing the β-1,3-glucanase gene on the load-bearing performance of the fibre cell wall and thus that of the retted fibres was rather negligible. That may be explained by the compensating impact of the various biochemical changes observed in the transgenic plants.

### Uronic acid content in the cell wall fractions

**Figure 5:**
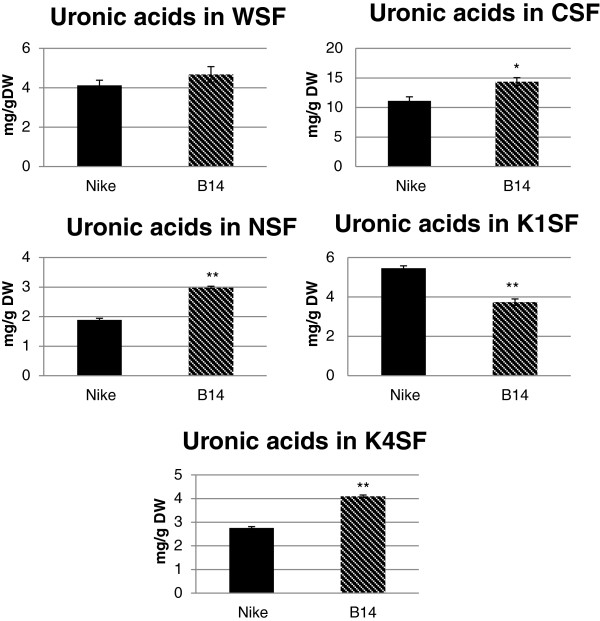
**Uronic acid contents in the cell wall fractions.** The uronic acid contents in five cell wall fractions (WSF – water-soluble fraction, CSF – CDTA-soluble fraction, NSF – Na_2_CO_3_-soluble fraction, K1SF – 1 M KOH-soluble fraction and K4SF – 4 M KOH-soluble fraction) were determined for fibres from transgenic flax line B14 (striped black bar) and non-transgenic control flax Nike (black bar). Analyses were performed as described in the Methods section. Data represents the mean values ± SD from three independent experiments. The significance of the differences between the means was determined using Student’s t test (* - P < 0.05, ** - P < 0.01).

During fractionation of the cell wall material, three fractions of pectin were obtained: the water-soluble fraction (WSF – pectin that is loosely associated with the cell wall); the CDTA-soluble fraction (CSF – pectin that is enriched in bound ions); and the Na_2_CO_3_-soluble fraction (NSF – pectin that is enriched in covalently bound ions). Two fractions of hemicellulose were also obtained: the 1 M KOH-soluble fraction (K1SF) and the 4 M KOH-soluble fraction (K4SF). The content of uronic acids (mainly galacturonic acid) was determined using spectrophotometry, as described in the Methods section. The data are presented in Figure 5.

The results obtained from the three fractions of pectin (WSF, CSF and NSF) revealed an increase in the uronic acid content in the fibres of transgenic line B14 compared to the values for the non-transgenic flax fibres. Statistically significant changes were observed in the CSF and NSF, whereas only a slight, non-significant increase was detected in the WSF. Galacturonic acid is the main component of pectic polysaccharides, so the CSF, which contains the highest amount of GalAc (14.5 mg/g DW), can be called the key fraction of a flax pectin.

The data obtained for the two hemicellulose fractions revealed a significant decrease in the uronic acid content in fibres from transgenic flax line B14 in K1SF and a significant increase in the fibres from transgenic flax in K4SF compared to the values for the control fibres.

### Monosaccharide composition in cell wall fractions

**Figure 6:**
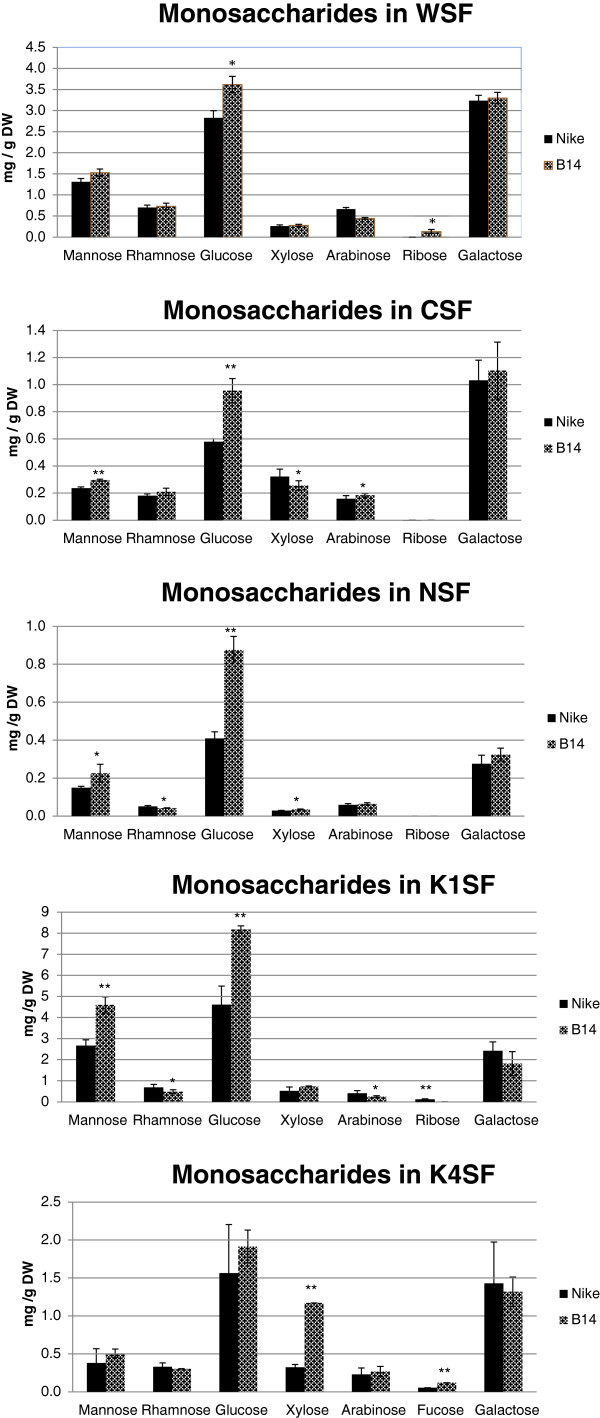
**Monosaccharide composition in the cell wall fractions.** UPLC analysis was performed of the monosaccharide contents in the cell wall fractions (WSF – water-soluble fraction, CSF – CDTA-soluble fraction, NSF – Na_2_CO_3_-soluble fraction, K1SF – 1 M KOH-soluble fraction and K4SF – 4 M KOH-soluble fraction) for fibres from transgenic flax line B14 (checked black bar) and non-transgenic control flax Nike (black bar). Analyses were performed as described in the Methods section. Data represents the mean values ± SD from three independent experiments. The significance of the differences between the means was determined using Student’s t test (* - P < 0.05, ** - P < 0.01).

The monosaccharide compositions of the three fractions of pectin (WSF, CSF and NSF) and the two fractions of hemicellulose (K1SF and K4SF) were analysed by UPLC. Figure 6 shows the statistically significant increase in glucose content in the three fraction of pectin in the fibres of transgenic line B14 relative to the contents for the control fibres The amount of galactose, the other major sugar, is increased in all the pectin fractions, but these changes were statistically insignificant. The only other sugar significantly changed in WSF fraction was ribose present however on a very low level. The more significant changes were observed for CSF fraction where increase in mannose and arabinose and decrease in xylose were found statistically significant. For NSF fraction statistically significant increase in mannose and xylose and lowering of rhamnose was observed.

Similarly the increase in glucose content was observed in hemicellulose fractions however those changes were statistically significant only for K1SF fractions. The mannose contents were significantly higher and rhamnose, arabinose and ribose significantly lower in K1SF fraction of the fibres from transgenic flax. A statistically significant increase in xylose and fucose content was found in the hemicellulose K4SF of the fibres of transgenic line B14 relative to the values for the non-transgenic plants. There were noticeable similarities in the changes in the contents of particular monosaccharides in the pectin and hemicellulose fractions. To better illustrate the changes in a pectin and hemicellulose composition the molar participation of particular monosaccharides in the pectin and hemicellulose fractions was calculated and is presented in Table 1. There are some significant differences between the monosaccharide composition of all of the fractions in the fibres of transgenic line B14 and the composition of the fibres of the control flax, mostly coinciding with the data presented as the quantity of monosaccharides (in mg of sugar per g of fibre). The most notable exception is in mannose whose molar ratio to other sugars is not statistically changed in all fractions except K1SF even though its general level is increased. The strongest and statistically significant changes in composition are observed for fractions NSF and K4SF. The data also confirmed that CSF is the main fraction of pectin, because its galacturonic acid content is higher than the content of other monosaccharides. K1SF and K4SF contain a considerable amount of xyloglucans compared to the pectin fractions.

**Table 1:** Molar composition of monosaccharides in the cell wall fractions

**Monosaccharide (mol/100 mol of total sugars)**										
**Fraction**		**Man**	**Rha**	**Glc**	**Xyl**	**Ara**	**Fuc**	**Rib**	**Gal**	**GalAc**
WSF	Nike	10.04 ± 0.87	5.88 ± 0.35	21.65 ± 0.63	2.38 ± 0.22	6.01 ± 0.31	0	0	24.80 ± 0.89	29.33 ± 1.63
	B14	10.41 ± 1.10	5.47 ± 0.61	24.67 ± 0.39*	2.27 ± 0.43	3.65 ± 1.08	0	1.67 ± 0.21	22.62 ± 1.22	29.62 ± 2.25
CSF	Nike	1.83 ± 0.10	1.54 ± 0.07	4.50 ± 0.25	2.99 ± 0.57	1.47 ± 0.21	0	1.06 ± 0.24	7.96 ± 1.18	78.80 ± 2.47
	B14	1.79 ± 0.41	1.39 ± 0.20	5.75 ± 0.51**	1.85 ± 0.26**	1.33 ± 0.11	0	0	6.70 ± 1.34	80.97 ± 2.15
NSF	Nike	5.56 ± 0.36	2.16 ± 0.05	14.78 ± 1.43	1.26 ± 0.08	2.56 ± 0.27	0	0	9.45 ± 0.83	65.07 ± 0.64
	B14	5.50 ± 1.71	1.11 ± 0.06**	20.77 ± 1.39**	1.02 ± 0.04**	1.85 ± 0.22**	0	0	7.40 ± 0.86*	67.41 ± 3.80
K1SF	Nike	15.76 ± 0.94	4.45 ± 0.65	26.99 ± 2.65	3.78 ± 1.38	3.04 ± 0.60	0	0.76 ± 0.18	14.68 ± 1.30	29.29 ± 3.47
	B14	23.17 ± 0.83**	2.74 ± 0.51**	41.01 ± 6.01**	4.44 ± 0.42	1.58 ± 0.23**	0	0*	9.48 ± 3.29*	17.53 ± 1. 84**
K4SF	Nike	6.71 ± 0.39	4.97 ± 0.58	22.30 ± 8.84	5.34 ± 0.39	3.76 ± 1.26	0.87 ± 0.04	0	20.42 ± 7.51	36.65 ± 2.40
	B14	5.19 ± 0.74	3.41 ± 0.13	19.92 ± 1.62	14.38 ± 0.41**	2.93 ± 0.59	1.46 ± 0.14	0	12.59 ± 0.01	39.62 ± 1.14

The amount of mol of the studied monosaccharides to 100 mol of monosaccharides in the cell wall fractions (WSF – water-soluble fraction, CSF – CDTA-soluble fraction, NSF – Na_2_CO_3_-soluble fraction, K1SF – 1 M KOH-soluble fraction and K4SF – 4 M KOH-soluble fraction) for fibres from transgenic flax line B14 and the non-transgenic control flax Nike. Man – mannose, Rha – rhamnose, Glc – glucose, Xyl – xylose, Ara – arabinose, Fuc- fucose, Rib – ribose, Gal – galactose and GalAc – galacturonic acid. Data represents the mean values ± SD from three independent experiments. The significance of the differences between the means was determined using Student’s t test (* - P < 0.05, ** - P < 0.01).

### Flax fibre callose content

**Figure 7:**
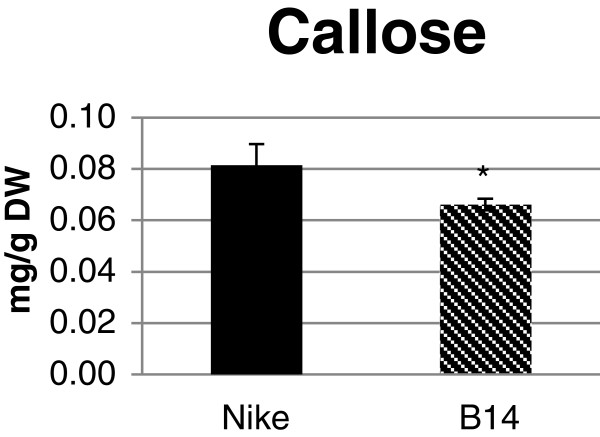
**Callose content in flax fibres.** The amount of callose was measured in the fibres of transgenic flax line B14 (striped black bar) and non-transgenic, control flax Nike (black bar). Analyses were performed as described in the Methods section. Data represent the mean values ± SD from five independent experiments.

Callose contents were determined using spectrofluorometry with β-1,3-glucan as the standard for preparing the calibration curve. The callose content was reduced in the fibres from transgenic flax B14 relative to that of the control flax fibres. The difference in the compound content came to 20% and was statistically significant (Figure 7).

### Phenolic acid, phenolic aldehyde and vitexin contents in the cell wall fractions of the cell wall

Phenolic acids (vanillic acid, coumaric acid and ferulic acid), phenolic aldehydes (syringic aldehyde and vanillin) and vitexin (C-glucosylated flavone) were detected in all of the cell wall fractions after alkaline hydrolysis, which indicated an ester bond with the monosaccharides that compose the pectin and hemicelluloses in the cell wall (Table 2). A statistically significant increase in the contents of vanillin, ferulic acid and vitexin, and a statistically non-significant increase in the contents of vanillic acid, coumaric acid and syringic aldehyde were detected in the CSF of the fibres from transgenic line B14 compared to the values for the control flax fibres. Slightly reduced levels of vanillic acid, coumaric acid and syringic aldehyde and slightly increased levels of ferulic acid, vanillin and vitexin were found in the WSF of the fibres from the transgenic flax. A significantly lower level of vanillic acid and syringic aldehyde and a lack of changes in the levels of the rest of phenolic compounds were detected in the NSF from the transgenic flax. The levels of all of the phenolic compounds with the exception of vitexin increased significantly in the hemicellulose K4SF and slightly in the hemicellulose K1SF of the fibres from transgenic flax compared to the levels for the non-transgenic plants. In the K1SF, the increases for coumaric acid, ferulic acid and vitexin were statistically significant.

**Table 2:** Phenolic compound contents in the cell wall fractions

**μg/g DW**							
**Fraction**		**Vanillic acid**	**Vanillin**	**Coumaric acid**	**Syringic aldehyde**	**Ferulic acid**	**Vitexin**
WSF	Nike	0.360 ± 0.048	0.108 ± 0.028	0.169 ± 0.065	0.152 ± 0.052	0.744 ± 0.142	0.757 ± 0.014
	B14	0.260 ± 0.007	0.150 ± 0.045	0.103 ± 0.036	0.09 ± 0.013	0.811 ± 0.336	0.930 ± 0.322
CSF	Nike	0.071 ± 0.003	0.009 ± 0.001	0.038 ± 0.004	0.016 ± 0.003	0.162 ± 0.006	0.213 ± 0.014
	B14	0.104 ± 0.032	0.016 ± 0.003*	0.055 ± 0.011	0.032 ± 0.018	0.295 ± 0.055*	0.326 ± 0.053*
NSF	Nike	0.258 ± 0.021	0.034 ± 0.009	0.150 ± 0.021	0.055 ± 0.009	0.635 ± 0.021	1.085 ± 0.085
	B14	0.201 ± 0.014*	0.043 ± 0.008	0.12 ± 0.014	0.042 ± 0.003*	0.622 ± 0.012	1.022 ± 0.074
K1SF	Nike	1.771 ± 0.115	1.196 ± 0.240	0.431 ± 0.041	1.212 ± 0.071	2.306 ± 0.176	3.349 ± 0.270
	B14	1.886 ± 0.530	1.917 ± 0.505	0.593 ± 0.032**	0.895 ± 0.11*	2.925 ± 0.281*	4.044 ± 0.361*
K4SF	Nike	0.477 ± 0.286	0.079 ± 0.01	0.046 ± 0.012	0.116 ± 0.048	0.145 ± 0.050	0.185 ± 0.047
	B14	1.597 ± 0.199*	0.336 ± 0.034**	0.127 ± 0.009**	0.370 ± 0.045**	0.563 ± 0.073**	0.256 ± 0.034

UPLC determination of the vanillic acid, coumaric acid, ferulic acid, vanillin, syringic aldehyde and vitexin contents in the cell wall fractions (WSF – water-soluble fraction, CSF – CDTA-soluble fraction, NSF – Na_2_CO_3_-soluble fraction, K1SF – 1 M KOH-soluble fraction and K4SF – 4 M KOH-soluble fraction) for fibres from transgenic flax line B14 and non-transgenic control flax Nike. Analyses were performed as described in the Methods section. Data represents the mean values ± SD from three independent experiments. The significance of the differences between the means was determined using Student’s t test (* - P < 0.05, ** - P < 0.01).

The content of phenolic compounds expressed as mmol per 100 mol of monosaccharides in the various cell wall fractions are shown in Table 3, giving a sense of the stoichiometry of the components of the analysed fractions. The highest level of phenolic compounds was measured in the hemicellulose K1SF in both the transgenic and control fibres. The calculated molar stoichiometry of phenolics and monosaccharides reveals that the hemicellulose K4SF accumulates a statistically significant excess of phenolic compounds detected in transgenic fibres. Vanillin and vanillic and phenolic acids were predominantly detected in this fraction. Since the monosaccharide content of the K4SF from both of the analysed fibre types differs mainly in terms of the xylose content, we speculate that this sugar unit is predominantly esterified by phenolic acids in the transgenic fibre. However, this needs to be verified.

**Table 3:** Molar phenolic compound content relative to monosaccharide content in cell wall fractions

**Mmol of phenolic / 100 mol monosaccharide in cell wall fractions**								
**Fraction**		**Vanillic acid**	**Vanillin**	**Coumaric acid**	**Syringic aldehyde**	**Ferulic acid**	**Vitexin**	**Sum of phenolic compounds**
WSF	Nike	2.95 ± 0.38	0.98 ± 0.25	1.42 ± 0.54	1.15 ± 0.40	5.28 ± 1.01	2.41 ± 1.39	14.18 ± 1.14
	B14	1.90 ± 0.05*	1.21 ± 0.69	0.78 ± 0.27	0.61 ± 0.09	5.15 ± 2.14	2.65 ± 0.92	12.31 ± 2.13
CSF	Nike	0.58 ± 0.03	0.08 ± 0.01	0.32 ± 0.04	0.12 ± 0.02	1.16 ± 0.05	0.69 ± 0.04	2.96 ± 0.16
	B14	0.68 ± 0.21*	0.11 ± 0.07	0.37 ± 0.07	0.19 ± 0.11	1.66 ± 0.31*	0.82 ± 0.13	3.84 ± 0.82
NSF	Nike	2.51 ± 0.32	0.37 ± 0.05	1.50 ± 0.08	0.50 ± 0.11	5.35 ± 0.85	4.11 ± 0.87	14.34 ± 2.07
	B14	1.65 ± 0.11*	0.39 ± 0.06	1.01 ± 0.09*	0.32 ± 0.08	4.42 ± 0.56	3.26 ± 0.90	11.05 ± 2.21
K1SF	Nike	11.30 ± 0.74	8.43 ± 0.69	2.82 ± 0.27	7.14 ± 0.42	12.74 ± 0.98	8.31 ± 0.67	50.75 ± 3.75
	B14	10.22 ± 2.87	11.48 ± 3.03	3.29 ± 0.18*	4.48 ± 0.55*	13.72 ± 1.32*	8.52 ± 0.76	51.71 ± 7.61
K4SF	Nike	7.27 ± 2.36	1.33 ± 0.78	0.71 ± 0.19	1.64 ± 0.68	1.92 ± 0.66	1.10 ± 0.28	13.98 ± 5.87
	B14	17.55 ± 2.19**	4.09 ± 0.41**	1.43 ± 0.10**	3.75 ± 0.45*	5.36 ± 0.70*	1.09 ± 0.14	33.28 ± 4.05**
All fractions	Nike	24.62 ± 5.56	11.19 ± 1.03	6.77 ± 0.93	10.55 ± 1.15	26.46 ± 1.85	16.61 ± 1.39	96.20 ± 8.98
	B14	32.00 ± 3.63	17.28 ± 2.41*	6.87 ± 0.40	9.35 ± 0.65	30.32 ± 2.37	16.36 ± 1.51	112.19 ± 5.15*

The amount of mmol of the studied phenolic compounds (vanillic acid, coumaric acid, ferulic acid, vanillin, syringic aldehyde and vitexin) to 100 mol of monosaccharides in the cell wall fractions (WSF – water-soluble fraction, CSF – CDTA-soluble fraction, NSF – Na_2_CO_3_-soluble fraction, K1SF – 1 M KOH-soluble fraction and K4SF – 4 M KOH-soluble fraction) for fibres from transgenic flax line B14 and non-transgenic control flax Nike. Data represents the mean values ± SD from three independent experiments. The significance of the differences between the means was determined using Student’s t test (* - P < 0.05, ** - P < 0.01).

### Antioxidant activity

**Figure 8:**
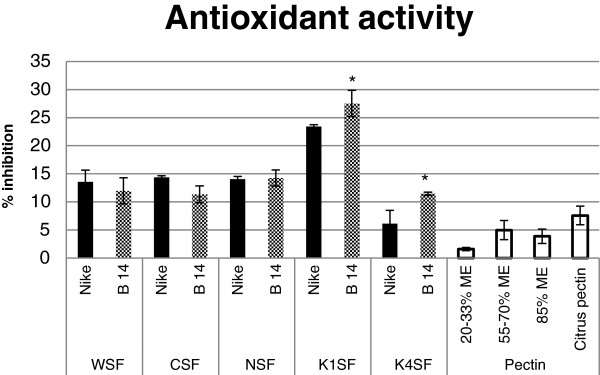
**Scavenging activity of the cell wall fraction against DPPH• radicals.** The antioxidant potential was measured in the cell wall fractions (WSF – water-soluble fraction, CSF – CDTA-soluble fraction, NSF – Na_2_CO_3_-soluble fraction, K1SF – 1 M KOH-soluble fraction and K4SF – 4 M KOH-soluble fraction) for fibres from transgenic flax line B14 (checked black bar) and non-transgenic control flax Nike (black bar) and compared with the values for commercially available citrus pectin with various ME values (20-33%, 55-70%, 85%) and standard citrus pectin at a concentration of 5 mg/ml (white bars). Analyses were performed as described in the Methods section. Data represents the mean values ± SD from three independent experiments. The significance of the differences between the means was determined using Student’s t test (* - P < 0.05).

The antioxidant activities of particular cell wall fractions were determined using the DPPH method. The free radical 2.2-diphenyl-1-picrylhydrazyl (DPPH•) is scavenged by antioxidants, and the percentage of inhibition could be measured as the decrease in absorbance at 515 nm. The scavenging activity of the pectin and hemicellulose fractions at a concentration of 5 mg/ml was compared with that of commercially available pectin at the same concentration (Figure 8). The antioxidant activity of commercial pectin was the lowest (5-7%). The percentage of inhibition of free radicals for the three pectin fractions (WSF, CSF and NSF) was between 12 and 15%. The antioxidant potential for transgenic fibre B14 was lower than that of the control, but these changes were statistically insignificant. The highest antioxidant potential was observed for the K1SF: from 23% inhibition for the control fibres and 27% for the B14 transgenic fibres. The K4SF displays an increase in antioxidant activity for the transgenic flax fibre (11% inhibition of free radicals) relative to the control flax fibre (6%). These data indicate that the hemicellulose K1SF has the best antioxidant potential, which correlates with the phenolic compound content (Table 3).

## Discussion

Flax (*Linum usitatissimum*) is a valuable source of oil rich in omega-3 fatty acids (mainly linolenic acid) and fibres containing antioxidant compounds. Flax fibres are mainly used by the textile industry, but the presence of valuable bioactive compounds means there is potential for biomedical applications as wound dressings and sutures [1].

The production of high-quality flax fibres is limited by several factors, such as plant productivity, the efficiency of straw processing (retting) and the contents and stoichiometry of biopolymers (cellulose, hemicellulose, pectin, lignin). The productivity of flax depends on abiotic (climate) and biotic (pathogens) factors. In our previous study, in order to improve productivity, flax plants with overexpression of the β-1,3-glucanase gene were generated and characterised by the increase in resistance to fungal infection [27]. This effect was expected because β-1,3-glucanase is responsible for the hydrolysis of β-1,3-glucans, the main compound of the fungal cell wall [25].

The aim of this study was to assess the fibres of fourth generation plants of this transgenic line that had been field-cultivated on a semi-technical scale in the 2010 growing season. The rationale behind the study was that fibre polysaccharides determine the technological properties of fibres and their industrial usefulness. β-1,3-glucanase is capable of endogenous β-1,3-glucan (callose) degradation and glucose derivatives are the main constituents of fibre polysaccharides, so the transgenic plants should display different properties.

There were no changes in the fibre yield from the transgenic flax that overproduces β-1,3-glucanase compared to the yield from the control plants. However, there were significant differences in both the quantity and quality of the main biopolymers that constitute the fibre. The genetic modification resulted in a slight increase in the cellulose content, a statistically significant increase in the pectin and hemicellulose contents, and a decrease in the lignin content in the fibres.

The biochemical data were confirmed by the IR analysis of the fibres. The integral intensities of the successive bands were assessed and differences in the cellulose, pectin, hemicellulose and lignin contents were found. The bands responsible for hydrogen bonds and thus polymer arrangement were also analysed. Fibres from the transgenic B14 line were characterised by a rise in band intensity in the 3000 to 3600 cm^-1^ range, resulting from different conformations of the intra- and inter-molecular hydrogen bonds in the cellulose. Also, the integral intensity of the 1236 cm^-1^ band was higher for B14 flax, suggesting an increase in the number of hydrogen bonds in the cellulose. Thus, the overexpression of the β-1,3-glucanase gene results in changes to the cellulose polymer organization, namely their closer packing in the fibres. One possible reason for the comparable fibre strength of the control and transgenic plants was the only slight change in the cellulose content. However, the reduced level of lignin affects the elastic properties of the fibre. The flexibility of fibres from the B14 line was elevated, as indicated by the lower Young’s modulus. Additionally, lignin plays a very important role during fungal infection. It is known that an increase in lignin content gives a higher resistance to fungal attacks [39,40]. However, some studies yielded results that indicate that plants are more resistant to fungal infection in spite of the reduced lignin content [41].

Significant changes in the pectin and hemicellulose contents observed in the fibres of the transgenic flax indicated the need for a detailed analysis of these polysaccharides, including an assessment of the monosaccharide composition and the phenolic acid content. The quantitative and qualitative contribution of pectin to the construction of the plant cell wall depends on the developmental stage [42] and the plant species [43,44]. The monosaccharide analysis revealed a higher content of galacturonic acid in all of the pectin fractions in the fibres from the transgenic flax, with the highest result in the CSF. Moreover, the fibres from B14 flax were characterised by a significant increase in the glucose content and an increase that was not always significant in the mannose and rhamnose content in all of the pectin fractions. Therefore, this genetic modification not only increased the quantity of pectin, but also changed its constituents. A similar tendency was observed in the two hemicellulose fractions from the fibres. Significantly elevated contents of glucose and mannose in the K1SF and xylose in the K4SF but slightly reduced contents of rhamnose and galactose in both fractions were detected in the fibres from the transgenic plants. There is a marked galacturonic acid content reduction in the hemicellulose K1SF in the transgenic plants.

The changes in pectin and hemicellulose composition (and thus predicted structure) are even better illustrated when molar ratio of participating sugars were calculated giving a clearer picture of stoichiometry of forming units. What is mostly visible is statistically significant increased percentage of glucose and insignificant increase in galactouronic acid content with decrease of contribution of all other sugars in pectin fractions. In case of hemicelluloses increase in glucose percentage is observed only for K1SF fraction. The increase in the content of predominant monosaccharides is noteworthy, particularly the significantly elevated glucose content, which raises questions about the underlying molecular mechanism. We reasoned that β-1,3-glucanase hydrolyses β-1,3-glucans not only from the fungal cell wall, but also flax callose, an endogenic β-1,3-glucan. Since the level of callose was reduced in the fibres from transgenic line B14 relative to the levels for the control fibres, we speculate that the excess of glucose units could be used as substrate for the synthesis of other wall polymers (pectin or hemicellulose) [45]. The detailed analysis of the wall constituents revealed significant differences in the content of phenolic compounds bound with the pectin and hemicellulose fractions. Three phenolic acids (ferulic, vanillin and coumaric acid), two phenolic aldehydes (vanillin and syringic aldehyde) and one flavonoid (vitexin) were identified in the pectin fractions isolated from fibres. The total content of phenolic compounds in the fibres from the transgenic plants was about 26% higher than in the fibres from the control plants. Of the wall fractions that were analysed, CSF and K4SF were characterised by a significant increase in the contents of most of the phenolic compounds. In K4SF, this is probably caused by the increase in xylose content in the transgenic flax fibres, as it was reported that phenolic acids bind preferentially to arabinoxylans [46,47]. In the remaining fractions, the quantities of particular compounds were different and depended on the fraction and the compound analysed.

The obtained results were reflected in the antioxidant properties of particular fractions. The highest antioxidant potential was shown by the K1SF of the fibres from the transgenic flax. It is worth noting that regardless of the similar quality of phenolic compounds in the WSF, NSF and K4SF, the hemicellulose fraction displayed the lowest ability to inhibit the reactive form DPPH•. It is speculated that besides the phenolic compound contents, the proper composition and structure of pectin might also affect the antioxidant properties [48]. It is interesting to note that the antioxidant activity of all of the wall fractions from flax fibres was statistically higher than that for commercially available pectin. The results confirmed that flax fibre pectin is potentially more beneficial than commercial citrus pectin and indicated the possibility of its biomedical application.

## Conclusion

Fibres from flax plants overexpressing the β-1,3-glucanase gene have an altered wall biopolymer composition. The contents of particular polysaccharides (cellulose, hemicellulose and pectin) were higher, but reduced lignin content was also observed. A decrease in the content of callose, which is a substrate for β-1,3-glucanase, was also found. It is thus suggested that the glucose units released from the callose are redirected to the production of other polysaccharides. The increase in the contents of monosaccharides, the main component of hemicellulose, is accompanied by an accumulation of phenolic compounds in this fraction. The reason for this accumulation is unknown.

The antioxidant potential of the pectin fractions could be predicted from the amount of phenolic compounds. The fibres from transgenic plants showed a higher antioxidant potential than those from the control plants, which suggests their potential usefulness as biomedical products.

In summary, this study shows for the first time that the β-1,3-glucanase gene is involved in plant polysaccharide and lignin metabolism and establishes its benefits in the production of improved flax fibres.

## Methods

### Plant material and retting

Flax seeds (*Linum usitatissimum* L., fibrous cultivar Nike) were obtained from the flax and hemp collection of the Institute of Natural Fibres of Poland. Fourth-generation plants of the transgenic line and control flax were field-cultivated in Wroclaw on a semi-technical scale during the 2010 growing season. The flax was harvested on the 107^th^ day of its growth. The field-grown plants were retted using the dew method, in which the plants are spread out in a field and left for at least 40 days. During this process, bacteria and fungi degrade the cell wall polysaccharides and middle lamella releasing the fibres from the stems [2]. The quantity of obtained fibres from whole straw is presented as % fibres in the straw (Figure 1).

### Transgenic plants

The plants were transformed using the plasmid pGAglubsens, containing cDNA encoding β-1,3-glucanase from potato (GenBank: AJ586575.1) under a 35S CaMV promoter and Nos terminator. Transgenic plants were pre-selected using PCR and selected using Western blot analysis. Three lines of transgenic plants were selected (B10, B11, B14) for *in vitro* testing. The B14 line was used for the field trials because it had the best productivity. None of the transgenic lines showed changes in plant height. The transgenic line B14 produced slightly more seeds than the non-transformed plants, while the transgenic lines B10 and B11 showed significant decreases in the yield of seeds [27].

### Infra-red spectrophotometry analysis

Infra-red spectrometry was used to determine the chemical composition and molecular structure of the fibres from the transgenic and control flax plants. The spectra were measured at room temperature using a Biorad 575C FT-IR spectrometer. Data were collected over a spectral range from 50 to 4000 cm^-1^ with a resolution of 2 cm^-1^. In the mid infra-red part of this range, samples were prepared in a KBr pellet. In the far infra-red part of this range, samples were suspended in Nujol.

### Mechanical analysis of fibre

Tensile tests of the retted fibres were conducted by means of a computer-driven Instron system (model 4452, High Wycombe, UK). The finest axially uniform 2.5- to 4.0-cm long filaments were carefully extracted by hand from the fibre bundles. The ends of the samples were glued and sandwiched between small plastic sheets using a cyanoacrylate adhesive and were pinched within serrated grips connected both to a load cell of 10 N capacity and to the immovable part of the testing machine. The adhesive was spread out in a very thin layer and allowed to partially dry and solidify before the fibre ends were attached to the plastic pieces to avoid undesirable impregnation of the free filament part with cyanoacrylate. Samples of about 10-mm gauge length were extended at a crosshead speed of 1 mm/min. The exact gauge length was determined for each fibre to an accuracy of 0.01 mm at a tensile load of 5 cN. The load displacement curve was recorded and used to evaluate the fibre tensile parameters with Bluehill 2 Software (Instron Co.).

Stiffness, measured as Young’ modulus, was calculated from the slope of a linear part of the load displacement curve (ΔF/Δx), using the formula:

$$E=\frac{\frac{\mathrm{\Delta F}}{\mathrm{\Delta x}}}{\frac{L}{\mathrm{Acw}}}$$

where L is the gauge length (corrected for each sample using Instron readings) and Acw is the effective cell wall cross-sectional area.

The maximum recorded load (F_max_) per initial cross-sectional area of cell wall material (Acw) was used as a tensile strength measure:

$$\sigma =\frac{F_{\max}}{\mathrm{Acw}}$$

The cross-sectional area of the cell wall material was evaluated using a gravimetric method [49,50] and the formula:

$$\mathrm{Acw}=\frac{m}{L\cdot \mathrm{dcw}}$$

where m is the weight of the fibre gauge length and dcw is the cell wall material density, assumed to equal 1540 kg m^-3^[51].

The energy to break (determined as the strain energy to maximum load) per unit mass of fibre sample and the relative extension at break were determined using Bluehill 2 software.

The sample weight within the gauge length, needed for the estimation of the cross-sectional area of the cell wall material, was determined soon after tensile measurements were completed, to an accuracy of 1 μg, using a highly precise microbalance (XP6, Mettler, Toledo).

### Cellulose content

The cellulose content was determined using the colourimetric method with anthrone reagent, as described by Ververis [52]. 15 mg dry, ground flax fibres were incubated with a mixture of nitric and acetic acid (1:8 v/v) for 1 h at 100°C and then centrifuged (5 min, 14000 rpm). The pellet was washed twice with water and then resuspended in 1 ml 67% H_2_SO_4_ (v/v). After mixing samples, cold anthrone reagent was added and the cellulose level in these samples was measured spectrophotometrically at 620 nm. Commercially available cellulose after hydrolysis was used for the calibration curve.

### Lignin content

The determination of the total lignin content was performed using the acetyl bromide method, as described Iiyama and Wallis [53]. 15 mg dry, ground flax fibres were heated for 2 h at 100°C, then 10 ml water was added to each sample, and the samples were heated for 1 h at 65°C with mixing every 10 min. Then the samples were filtered through a GF/A glass fibre filter and rinsed three times with each of the following solutions: water, ethanol, acetone and diethyl ether. The filters were placed in glass vials and heated overnight at 70°C. After that, 25% acetyl bromide (2.5 ml) in acetic acid was added and the vials were placed at 50°C for 2 h. The cooled samples were mixed with 10 ml of 2 N sodium hydroxide and 12 ml of acetic acid. After incubating in RT overnight, the lignin content was measured at 280 nm. Coniferyl alcohol was used to prepare a calibration curve.

### Isolation and fractionation of the cell wall polysaccharides

The isolation and fractionation of the cell wall components was performed using a modified version of the method described by Manganaris [54] and Vincente [55].

Fibres from transgenic and non-transgenic flax (1 g dry, ground plant tissue) were boiled in 96% ethanol for 30 min to inactivate the enzymes, extract the low molecular weight components and prevent autolysis. The material was filtered with a Whatman GF/C filter and then sequentially washed with 80% ethanol, chloroform:methanol (1:1 v/v) and acetone, and allowed to dry at 37°C to yield an alcohol-insoluble residue (AIR).

All the AIR obtained from each sample was suspended in 20 ml of water and then stirred at RT for 12 h. After the centrifugation (6000 × g, 4°C, 10 min) the pellet was washed with water and both supernatants were collected for water-soluble fraction (WSF) analysis. The remaining material was resuspended in 50 mM CDTA (trans-1,2-diaminocyclohexane-N,N,N,N-tetraacetic acid) at pH 6.5 and stirred (RT, 12 h). After the centrifugation and wash (as above), the extracted solutions were collected and designated the CDTA-soluble fraction (CSF). The pellet was resuspended in 50 mM Na_2_CO_3_ with 20 mM NaBH_4_, stirred at 4°C for 12 h and washed, and then supernatants were neutralised with glacial acetic acid. These samples were designed the Na_2_CO_3_-soluble fraction (NSF). The remaining pelleted material was resuspended in 1 M KOH with 20 mM NaBH_4_, stirred at RT for 12 h and washed, and then supernatants were neutralised with HCl to yield the 1 M KOH-soluble fraction (K1SF). The same activity was performed with 4 M KOH to obtain the 4 M KOH-soluble fraction (K4SF). Supernatants from the CSF, NSF, K1SF and K4SF were extensively dialysed against water (with a 3.5-kDa cut off) and all of the fractions were additionally lyophilised before use.

### Uronic acid measurement

The content of pectin was determined using the biphenyl method [56] after hydrolysis of the polysaccharides in sulphuric acid [57]. The samples were suspended in 0.1 ml sulphuric acid and stirred in an ice bath for 5 min. Sequentially, 0.1 ml sulphuric acid, 0.05 ml water, 0.05 ml water and 0.7 ml water were added, with stirring between additions. The diluted material was centrifuged for 10 min at 2000 × g at RT, and 0.1 ml of the supernatant was taken and added to a 10-μl 4 M sulphamic acid/potassium sulphamate solution at pH 1.6. Then 0.6 ml of 75 mM Na_2_B_4_O_7_ in sulphuric acid was added for the reaction. The samples were shaken and incubated at 100°C for 20 min. After cooling, 20 μl of m-hydroxy-biphenyl (0.15%) in 0.5% NaOH was added to each sample, and they were incubated at RT for 10 min. The pectin content was measured with a spectrophotometer at 525 nm. Galacturonic acid was used for the calibration curve.

### Monosaccharide identification by UPLC

The hydrolysis of polysaccharides, derivatization procedure and UPLC analysis were performed using a modified version of the method describe by Lv [58] and Yang [59]. The lyophilized tissue samples (10 mg) were hydrolysed with 4 M TFA (500 ml) for 8 h at 110°C, and then cooled and centrifuged (5 min, 1000 rpm, RT). The pellet was discarded and the supernatant was dried under nitrogen and then dissolved in distilled water (1 ml).

In order to determine their monosaccharide composition, the samples (50 μl) were derivatised by incubation for 60 min at 70°C with 0.3 M NaOH (50 μl) and 0.5 M PMP in methanol (50 μl). After cooling, 0.3 M HCl (50 μl) was added to each sample and they were washed three times with chloroform. Each sample was filtered before UPLC analysis.

The samples were analysed on a Waters Acquity UPLC system with a 2996 PDA detector, using Acquity UPLC column BEH C18, 1.0 × 100 mm, 1.7 μm. The mobile phase was A = 50 mM CH_3_COONa, pH 6.3 with 0.04% TEA and B = acetonitrile with 0.04% TEA, in a gradient flow: 1 min at 96% A/4% B; 5–11 min gradient to 89% A/11% B; 12–13 min gradient to 0% A/100% B; and 14 min gradient to 96% A/4% B with a 0.05-ml/min flow rate. The content was measured at 250 nm.

### Total pectin and total hemicellulose contents

The content of total pectin was estimated as the sum of the uronic acids and other monosaccharides from the three pectin fractions (WSF, CSF and NSF).

The total hemicellulose content was calculated in the same way for the other two fractions (K1SF and K4SF).

### Callose content

The determination of the callose content in the flax fibres was performed using a modification of a method described by Hirano [60]. 20 mg dry, ground flax fibres were washed once with 96% ethanol and three times with 20% ethanol. Then, 1 ml of 1 M NaOH was added to the washed tissue, and to solubilise the callose, the tubes were heated at 80°C for 15 min. After the centrifugation (15 min, 10000×g), the supernatant was ready for the callose determination. 0.2 ml of supernatant, 0.4 ml of 0.1% (w/v) aniline blue, 0.21 ml of 1 M HCl and 0.59 ml of 1 M glycine-NaOH buffer (pH 9.5) were mixed and incubated for 20 min at 50°C, and then 30 min at room temperature. The callose content was quantified spectrofluorometrically at excitation and emission wavelengths of 393 and 484 nm, respectively. Curdlan (β-1,3-glucan) was used to prepare a calibration curve.

### Phenolic compound extraction and measurement by UPLC

The lyophilized samples (20–100 mg) from each fraction of the cell wall were extracted three times with methanol using an ultrasonic bath (15 min). After centrifugation (5 min, 5000 rpm, RT), the supernatant was collected and the pellet was hydrolysed using 2 M NaOH (2 ml) in the dark. Then, the pH was adjusted to 3.0, and the samples were extracted three times with ethyl acetate and centrifuged (1 min, 5000 rpm, RT). The supernatant was dried, the pellet was resuspended in methanol (0.2 ml) and the samples were analysed on a Waters Acquity UPLC system with a 2996 PDA detector, using an Acquity UPLC column BEH C18, 2.1 × 100 mm, 1.7 μm. The mobile phase was A = 0.1% formic acid and B = acetonitrile, in a gradient flow: 1 min at 95% A/5% B; 12 min gradient to 70% A/30% B; 15 min gradient to 0% A/100% B; and 17 min 95% A/5% B with a 0.1 ml/min flow rate. The detection of coumaric and ferulic acid, syringic aldehyde, vanillin and vitexin was done at 320 nm and that of vanillic acid at 280 nm.

### Antioxidant activity

The antioxidant activity was assessed as described by Brand-Williams with some modifications [61]. 10 mg of lyophilised samples from each cell wall fraction were resuspended in 1 ml of water. In a similar way, standards of pectin with different degrees of methylation were prepared. A 1 ml solution of 0.1 mM DPPH (2,2-diphenyl-1-picrylhydrazyl) in water:methanol (1:1) was mixed with 50 μl of sample. After 6 h, the absorbance was measured at 515 nm. Ferulic acid was used as a positive control.

The inhibition of DPPH• radicals of the cell wall fraction was calculated according to the equation:

$$\left(\%\right)\mathrm{inhibition}=\left[1-\left(A_{\mathrm{sample}}515\;\mathrm{nm}/A_{\mathrm{control}}515\;\mathrm{nm}\right)\right]\times 100$$

### Statistical analysis

All of the experiments were independently repeated at least three times. The results are presented as the averages of independent replicates ± standard deviations. Statistical analyses were performed using Statistica 7 software (Statsoft, USA). The significance of the differences between the means was determined using Student’s t test.

## Abbreviations

CSF: CDTA-soluble fraction;DPPH: 2.2-diphenyl-1-picrylhydrazyl;GalAc: Galacturonic acid;K1SF: 1 M KOH-soluble fraction;K4SF: 4 M-KOH soluble fraction;NSF: Na_2_CO_3_-soluble fraction;WSF: Water-soluble fraction

## Competing interests

The authors declare that they have no competing interests.

## Authors’ contributions

WW performed all the biochemical experiments and statistical analyses and wrote the manuscript. AK designed the experiments, carried out the UPLC analysis and participated in writing the manuscript. LD performed the infra-red spectrophotometry analysis and participated in writing the IR-related section of the manuscript. JH participated in the infra-red spectrophotometry analysis. JŻ performed the mechanical analysis. JS participated in study design and coordination. All of the authors read and approved the final version of the manuscript.
